# Supplementary material for: Combine with RNA-seq Reveals the Effect of Melatonin in the Synthesis of Melanin in Primary Melanocytes of Silky Fowls Black-Bone Chicken
Source: Genes (Basel). 2023 Aug 18;14(8):1648. doi: 10.3390/genes14081648 (PMC10454632; doi:10.3390/genes14081648)
Supplement: Supplementary file 1 [file genes-14-01648-s001.zip › Table S1.pdf]

Table S1. Summary statistics for quality control of the sequencing data.

Sample	Raw Reads	Raw Bases(G)	Clean Reads	Clean Bases(G)	Q20(%)	Q30(%)	GC Content(%)
Cont_1	45,171,176	6.78	43,981,358	6.60	97.20	92.78	51.23
Cont_2	45,356,912	6.80	44,297,264	6.64	97.31	93.00	51.12
Cont_3	44,456,700	6.67	43,268,666	6.49	97.11	92.55	51.35
MT_1	44,173,684	6.63	43,203,874	6.48	97.27	92.83	50.44
MT_2	45,368,272	6.81	44,300,540	6.65	97.49	93.39	51.00
MT_3	43,558,374	6.53	42,560,828	6.38	97.50	93.36	50.56
