# Supplementary material for: Combine with RNA-seq Reveals the Effect of Melatonin in the Synthesis of Melanin in Primary Melanocytes of Silky Fowls Black-Bone Chicken
Source: Genes (Basel). 2023 Aug 18;14(8):1648. doi: 10.3390/genes14081648 (PMC10454632; doi:10.3390/genes14081648)
Supplement: Supplementary file 1 [file genes-14-01648-s001.zip › Table S2.pdf]

Table S2. Summary statistics of clean reads mapped to the reference genome.

Sample	Total Reads	Total Mapped Reads(%)	Unique Mapped Reads(%)	Exon(%)	Intron(%)	Intergenic (%)
Cont_1	43,981,358	91.18	89.40	78.58	8.64	12.78
Cont_2	44,297,264	91.35	89.41	77.83	9.44	12.73
Cont_3	43,268,666	91.24	89.21	77.54	9.99	12.47
MT_1	43,203,874	91.61	89.85	78.66	8.29	13.04
MT_2	44,300,540	91.51	89.69	78.26	9.02	12.73
MT_3	42,560,828	91.85	89.87	78.61	8.09	13.30
